# Accelerating universal health coverage: a call for papers

**DOI:** 10.2471/BLT.19.230904

**Published:** 2019-03-01

**Authors:** Woranan Witthayapipopsakul, Anond Kulthanmanusorn, Walaiporn Patcharanarumol, Rapeepong Suphanchaimat, Churnrurtai Kanchanachitra, Agnès Soucat, Viroj Tangcharoensathien

**Affiliations:** a International Health Policy Program, Ministry of Public Health, Tiwanon Road, Nonthaburi 11000, Thailand.; b Institute for Population and Social Research, Mahidol University, Nakhon Pathom, Thailand.; C Department of Health Systems, Governance and Financing, World Health Organization, Geneva, Switzerland.

In 2015, all United Nations (UN) Member States committed to achieving universal health coverage (UHC) through the health-related sustainable development goal (SDG 3). Achieving UHC would enable all people to afford the right to health throughout their life course.^1^ A recent study shows that people living in countries that achieve UHC have longer life expectancy at birth and healthier life expectancy than those living without UHC.^2^ UHC also has benefits that go beyond health. High out-of-pocket costs can lead to catastrophic health expenditure and impoverishment.^3^ Good health is one of the pillars of human development; it enables people to pursue their education and their personal and professional goals, contributes to alleviating poverty and reducing socioeconomic inequity.^4^ UHC is a human capital investment and a comprehensive public health policy that governments should pursue.

Several UN resolutions have called for accelerating progress towards equitable access to health services; a UN high-level meeting on UHC will be held in 2019 to this effect,^5^ while December 12 has been proclaimed International Universal Health Coverage Day.^6^ The World Health Organization’s 13th General Programme of Work aims to achieve a triple-billion target: one billion more people benefiting from UHC, one billion more people protected from health emergencies and one billion more people enjoying better health and well-being. UHC contributes to these three targets.^7^

However, this global commitment has not yet been matched with significant progress. As a baseline, in 2015, half of the world’s population was unable to access essential health services. The *2017 Global UHC Monitoring Report* indicated that the average service coverage index was only 64 out of 100, with great variation across countries and regions. The index was highest in East Asia, North America and Europe (77/100) and lowest in sub-Saharan Africa (42/100) followed by South Asia (53/100).^3^ With the current pace of progress in low- and middle-income countries, the UHC target will not be attained by 2030. Sustained political and financial commitments are therefore needed to accelerate progress towards UHC.

Achieving UHC requires mobilization of adequate resources and equitable, transparent and efficient allocation. Expanding the three dimensions of UHC; population coverage, service coverage and financial protection, requires evidence-informed policies and implementation capacities. Increasing the number of people covered by insurance does not always lead to better access to services or greater financial protection, as seen in China, Indonesia, Malaysia, the Philippines and Sri Lanka.^3^^,^^8^

The way public and private providers are paid influences the efficiency of health systems. Fee-for-service can induce over-provision of health care and high out-of-pocket costs.^9^^,^^10^ Strategic purchasing through close-ended payments does not stimulate overuse of health resources and improves the efficiency of health systems and population health outcomes.^11^ These experiences highlight the need for proper system design, strategic purchasing within health financing policies, good governance and effective monitoring to achieve UHC goals, more efficient health systems and equity in access.

In 2020, the world will still have a decade to harness global momentum and advance progress towards UHC by 2030. The *Bulletin of the World Health Organization* will publish a theme issue on accelerating progress towards UHC to encourage learning and information sharing on this dimension of the SDGs.

The issue will explore policy options and country experiences on how to expand population coverage, service coverage and financial protection. We welcome manuscripts that capture knowledge and experience in addressing bottlenecks and root causes of stagnation that hamper successful UHC advancement. We encourage analysis of breakthroughs in health systems that have been conducive to rapid expansion of coverage. Papers should focus on, for example, implementation science in health systems, innovative health financing, strategic purchasing, UHC and primary health care, the role of the private sector, policy coherence across government levels (particularly in decentralized health systems), the role of innovative technology and the design and use of health information. Best practices in good governance for health, based on transparency and accountability, would also be useful to learn how vested interests that hamper progress towards UHC are countered in different socioeconomic and political contexts. Comparative cross-country analyses are encouraged.

The deadline for submission is 15 June 2019. Manuscripts should be submitted in accordance with the *Bulletin*’s guidelines for contributors (available at: http://www.who.int/bulletin/volumes/96/1/18-990118/en/) and the cover letter should mention this call for papers. 

This theme issue will be launched at the Prince Mahidol Award Conference on Universal Health Coverage in January 2020.
